# Supplementary material for: Nutritional Intake, White Matter Integrity, and Neurodevelopment in Extremely Preterm Born Infants
Source: Nutrients. 2021 Sep 27;13(10):3409. doi: 10.3390/nu13103409 (PMC8539908; doi:10.3390/nu13103409)
Supplement: Supplementary file 1 [file nutrients-13-03409-s001.zip › Hortensius_nutrition_DTI_neurodevelopment_Supplemental_Figure_1_description_240921.pdf]

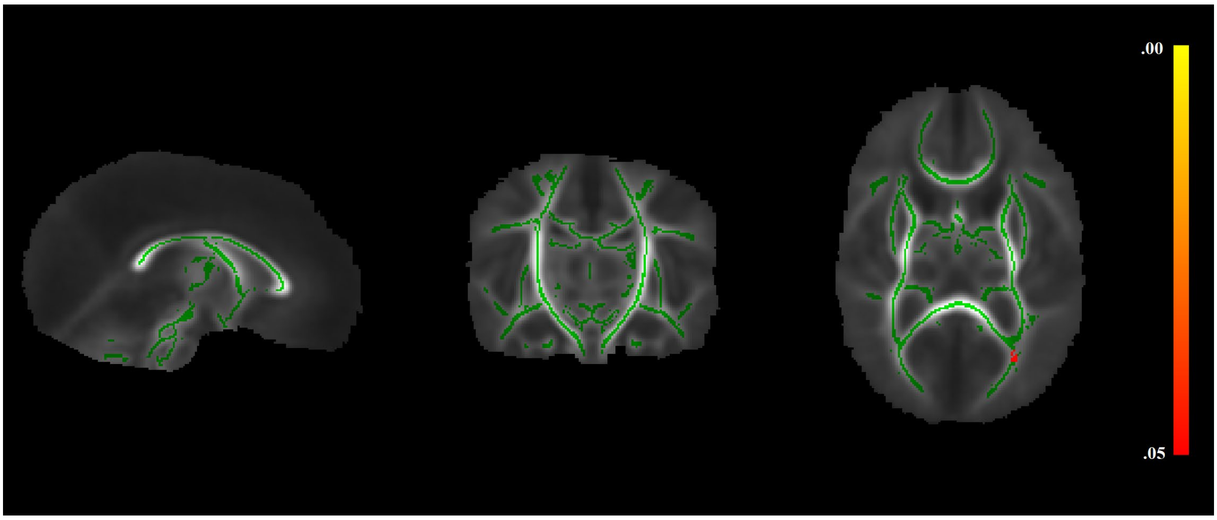

Figure S1. Cumulative enteral protein intake was associated with higher FA in the left posterior thalamic radiation. Significant voxels (red-yellow; color bar indicates *p*-value) are presented on top of the mean FA skeleton (green) and are viewed from a sagittal (left), coronal (middle), and axial (right) perspective.
