# Supplementary material for: Nutritional Intake, White Matter Integrity, and Neurodevelopment in Extremely Preterm Born Infants
Source: Nutrients. 2021 Sep 27;13(10):3409. doi: 10.3390/nu13103409 (PMC8539908; doi:10.3390/nu13103409)
Supplement: Supplementary file 1 [file nutrients-13-03409-s001.zip › Hortensius_nutrition_DTI_neurodevelopment_Supplemental_Table_1_240921.pdf]

Table S1. Baseline characteristics of the included and excluded children

	**DTI analysis**		**2 years corrected age**		**5.9 years chronological age**	
	**Included (n=123)**	**Excluded (n=55)**	**Included (n=161)**	**Excluded (n=17)**	**Included (n=154)**	**Excluded (n=24)**
Male (%)	57 (46)	27 (49)	75 (47)	9 (53)	72 (47)	12 (50)
Gestational age (weeks) (median Q1; Q3))	26 + 3 (25 + 6; 27 + 2)	26 + 3 (25 + 3; 27 + 2)	26 + 3 (25 + 6; 27 + 1)	27 + 1 (25 + 3; 27 + 2)	26 + 3 (25 + 6; 27 + 2)	26 + 4 (25 + 5; 27 + 2)
Birth weight (g) (median (Q1; Q3))	880 (784; 1000)*	835 (698; 913)*	870 (750; 995)	840 (695; 1025)	870 (750; 1000)	858 (710; 963)
Birth weight Z-score (mean (SD))	0.39 (0.88)*	0.01 (0.92)*	0.30 (0.91)	0.08 (0.89)	0.28 (0.90)	0.24 (0.96)
SGA (<10th percentile) (%)	5 (4)	5 (9)	8 (5)	2 (12)	8 (5)	2 (8)
Multiplicity (%)	36 (30)	15 (29)	48 (31)	3 (19)	52 (34)	4 (17)
Apgar 5 min (median (Q1; Q3))	8 (7; 8)	8 (7; 9)	8 (7; 9)	8 (6; 8)	8 (7; 9)	8 (5; 8)
Days parental nutrition (median (Q1; Q3))	13 (10; 17)	13 (10; 17)	12 (10; 17)	14 (13; 18)	12 (10; 17)	14 (11; 20)
>7 days of ventilation (%)	62 (50)	28 (51)	83 (52)	7 (41)	79 (51)	11 (46)
Abdominal surgery (%)	10 (8)	5 (9)	14 (9)	1 (6)	14 (9)	1 (4)
Severe brain injury (%)	14 (11)	4 (7)	17 (11)	1 (6)	15 (10)	3 (13)
Sepsis (%)	48 (39)	23 (42)	62 (39)	9 (53)	59 (38)	12 (50)

*DTI = diffusion tensor imaging; SGA = small for gestational age; *p<0.05*
