# Supplementary material for: Nutritional Intake, White Matter Integrity, and Neurodevelopment in Extremely Preterm Born Infants
Source: Nutrients. 2021 Sep 27;13(10):3409. doi: 10.3390/nu13103409 (PMC8539908; doi:10.3390/nu13103409)
Supplement: Supplementary file 1 [file nutrients-13-03409-s001.zip › Hortensius_nutrition_DTI_neurodevelopment_Supplemental_Table_2_240921.pdf]

Table S2. Baseline characteristics of the children included in cohort A and B

	DTI analysis		2 years corrected age		5.9 years chronological age	
	**Cohort A (N = 63)**	**Cohort B (N = 60)**	**Cohort A (N=95)**	**Cohort B (N=66)**	**Cohort A (N=92)**	**Cohort B (N=62)**
Male (%)	25 (40)	32 (53)	42 (44)	33 (50)	40 (44)	30 (48)
Gestational age (weeks) (median (Q1; Q3))	26 + 3 (25 + 6; 27 + 1)	26 + 4 (26 + 0; 27 + 2)	26 + 3 (25 + 6; 27 + 2)	26 + 3 (25 + 6; 27 + 0)	26 + 3 (26 + 5; 27 + 2)	26 + 5 (26 + 0; 27 + 1)
Birth weight (g) (median (Q1; Q3))	880 (792; 1000)	878 (784; 1005)	870 (750; 998)	875 (780; 958)	865 (749; 991)	878 (781; 1000)
Birth weight Z-score (mean (SD))	0.53 (0.85)	0.25 (0.89)	0.31 (0.92)	0.27 (0.90)	0.31 (0.93)	0.23 (0.87)
SGA (<10th percentile) (%)	2 (3)	3 (5)	5 (5)	3 (5)	5 (5)	3 (5)
Multiplicity (%)	20 (33)	16 (27)	27 (30)	21 (32)	28 (32)	20 (32)
Apgar 5 min (median (Q1; Q3))	8 (7; 9)	8 (6; 8)	8 (7; 9)*	8 (7; 8)*	8 (7; 9)	8 (7; 8)
Days parental nutrition (median (Q1; Q3))	12 (9; 15)	13 (10; 18)	12 (9; 16)	13 (10; 18)	12 (9; 16)	12 (10; 17)
>7 days of ventilation (%)	32 (51)	30 (50)	48 (51)	35 (53)	47 (51)	32 (52)
Abdominal surgery (%)	5 (8)	5 (8)	9 (10)	5 (8)	9 (10)	5 (8)
Severe brain injury (%)	8 (13)	6 (10)	10 (11)	7 (11)	9 (10)	6 (10)
Sepsis (%)	26 (41)	22 (37)	39 (41)	23 (35)	37 (40)	22 (35)

*DTI = diffusion tensor imaging; SGA = small for gestational age; *p<0.05*
