# Supplementary figures and images for: Nutritional Intake, White Matter Integrity, and Neurodevelopment in Extremely Preterm Born Infants
Source: Nutrients. 2021 Sep 27;13(10):3409. doi: 10.3390/nu13103409 (PMC8539908; doi:10.3390/nu13103409)

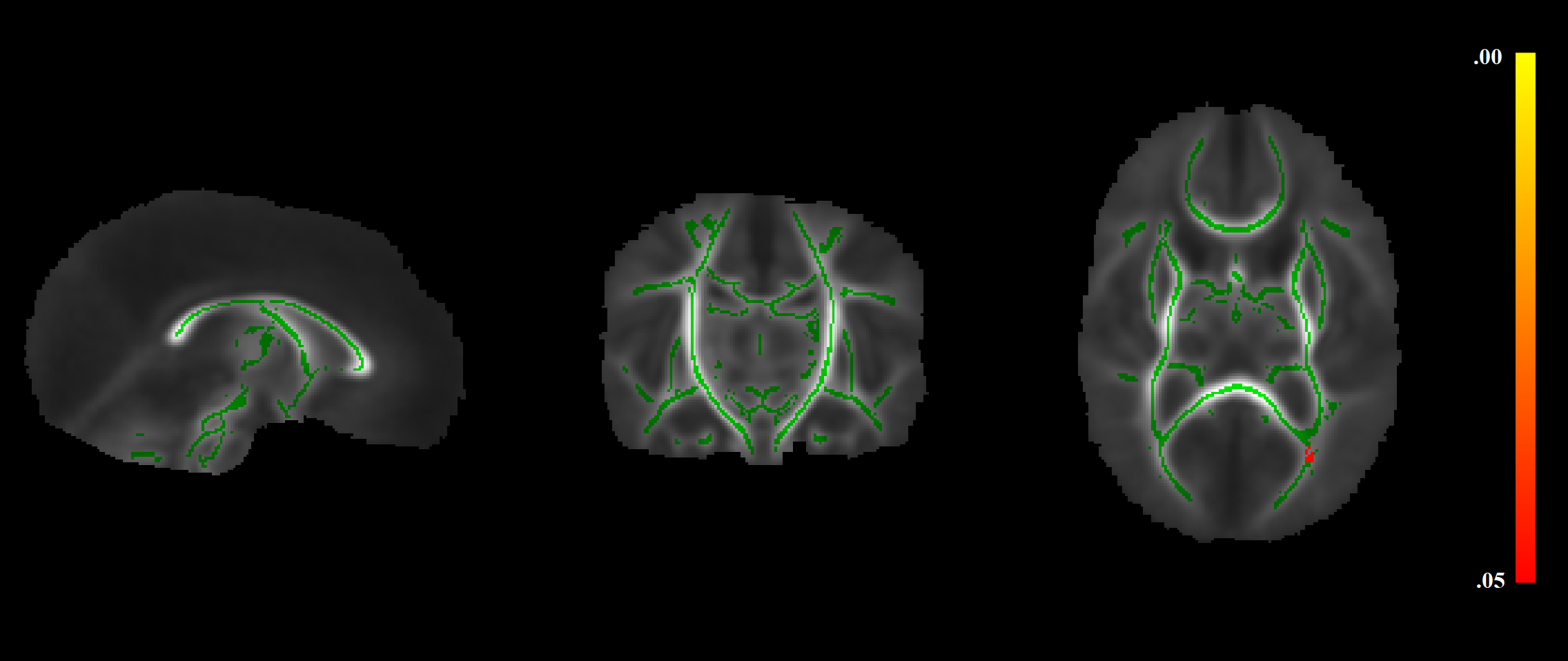

Supplement: Supplementary file 1 [file nutrients-13-03409-s001.zip › Hortensius_nutrition_DTI_neurodevelopment_Supplemental_Figure_1_250821.png]
